# Systematic review and meta-analysis of the efficacy and safety of adjunctive use of tirofiban in patients treated with endovascular therapy for acute ischemic stroke at different embolic sites

**DOI:** 10.1097/MD.0000000000035091

**Published:** 2023-10-06

**Authors:** Chenxi Liu, Xun Yang, Mingsu Liu, Jinping Wang, Guangqing Li

**Affiliations:** a Department of Neurology, The Frist Affiliated Hospital of Chongqing Medical University, Chongqing, China; b Department of Neurology, Hechuan District People’s Hospital, Chongqing, China; c Department of Neurology, Zhongshan Hospital Affiliated to Fudan University, Shanghai, China; d Department of Neurology, Chongqing University Central Hospital, Chongqing, China.

**Keywords:** acute ischemic stroke, endovascular treatment, tirofiban

## Abstract

**Background:**

The use of tirofiban as an adjunct to endovascular therapy (EVT) for acute ischemic stroke has been controversial. We aimed to assess the differences in safety and efficacy of EVT adjuvant to tirofiban in patients with anterior circulation stroke (ACS) and posterior circulation stroke (PCS).

**Methods:**

We systematically searched Pubmed, Embase, Cochrane Library, and Web of Science. Cohort studies and randomized controlled trials that compared treatment with tirofiban combined with EVT and EVT alone were included in our meta-analysis. The safety outcomes were symptomatic intracranial hemorrhage, and 3-month mortality. The efficacy outcomes were good functional outcome, excellent functional outcome, and successful recanalization (mTICI ≥ 2b). We performed subgroup analyses of anterior and posterior circulation strokes.

**Results:**

We included 15 studies with 4608 patients. For safety outcomes, tirofiban significantly reduced 3-month mortality in the ACS subgroup (odd ratio [OR] = 0.80, 95% confidence interval [CI] = 0.65–0.98, *P* = .03) without increasing the rate of symptomatic intracranial hemorrhage (OR = 1.12, 95% CI = 0.88–1.44, *P* = .35). In the PCS subgroup, tirofiban significantly reduced 3-month mortality (OR = 0.63, 95% CI = 0.50–0.80, *P* = .0001) and symptomatic intracranial hemorrhage (OR = 0.60, 95% CI = 0.37–0.95, *P* = .03). For efficacy outcomes, in the ACS subgroup, tirofiban significantly improved good functional outcome (OR = 1.24, 95% CI = 1.06–1.45, *P* = .008) but did not improve recanalization (OR = 1.17, 95% CI = 0.93–1.47, *P* = .17) and excellent functional outcome (OR = 1.19, 95% CI = 0.97–1.46, *P* = .10). In the PCS subgroup, tirofiban significantly improved recanalization rate (OR = 1.94, 95% CI = 1.43–2.65, *P* < .0001) and did not improve good functional outcome (OR = 1.03, 95% CI = 0.81–1.30, *P* = .81) and excellent functional outcome (OR = 0.84, 95% CI = 0.58–1.20, *P* = .34).

**Conclusion:**

In acute ischemic stroke patients undergoing EVT, tirofiban improves good functional outcomes in ACS patients and increases recanalization rates in PCS patients on the 1 hand, reduces mortality, and does not increase the risk of symptomatic intracranial hemorrhage on the other. Tirofiban is safe and effective in both anterior circulation stroke and posterior circulation stroke patients undergoing EVT. More large multicentre randomized controlled studies are needed in the future.

## 1. Introduction

Acute ischemic stroke is characterized by high morbidity, disability, and mortality.^[1]^ In recent years, several large randomized controlled clinical trials (RCTs)^[2–6]^ have demonstrated that endovascular treatment (EVT) has a higher rate of recanalization and a longer treatment window for acute anterior circulation stroke than drug therapy alone, and is effective in improving patient prognosis and reduce mortality. However, the procedure can cause endothelial damage, allowing platelet aggregation and thrombosis, with some risk of re-embolism or hemorrhage.^[7]^ Tirofiban is a highly selective GP IIb/IIIa receptor antagonist that mechanistically blocks the final common pathway of platelet aggregation and thrombosis.^[8]^ Neuro interventionalists are now evaluating tirofiban as an adjunct to EVT in patients with acute ischemic stroke (AIS) patients.

It is worth noting that although EVT has been used for several years in patients with acute stroke in the anterior circulation, the evidence for the use of EVT in patients with acute stroke in the posterior circulation is not sufficient. Past best studies,^[9]^ basics studies^[10]^ and recent basilar studies,^[11]^ baoche studies,^[12]^ and attention studies^[13]^ have reached different conclusions regarding the efficacy of EVT in posterior circulation stroke. In addition, previous studies have suggested that patients with acute stroke in the posterior circulation have more typical vascular-related risk factors and a higher subgroup proportion of atherosclerotic stroke.^[14,15]^ This may also increase the difference in efficacy of tirofiban in patients with EVT in the anteroposterior circulation stroke.

The previous Tang et al^[16]^ studies were divided into 2 subgroups of anterior circulation and posterior circulation stroke according to different sites of embolization and analyzed the differences between tirofiban in symptomatic intracranial hemorrhage (sICH) and good functional outcome in EVT patients, and both indicators yielded negative results. Tang study was included in studies before 2019 when there were no RCTs with high evidence-based evidence for vertebrobasilar thrombectomy when most guidelines were highly controversial about the implementation of posterior circulation stroke and few studies on perioperative antiplatelet in vertebrobasilar thrombectomy. Therefore, Tang study did not analyze the specific causes in detail but only listed the results. Second, for the safety and efficacy outcomes after thrombectomy, there are many indicators, such as 3-month mortality, recanalization rate, and excellent functional outcome, which are often closely related to clinical prognosis. Third, the effect of the regimen of oral antiplatelet drugs in the control group is also a factor to consider. Therefore, we conducted a comprehensive meta-analysis of the efficacy of tirofiban in patients with anterior and posterior circulation stroke, analyzed the reasons for the differences, and used this as a reference for subsequent treatment.

## 2. Materials and methods

This meta-analysis was conducted in accordance with PRISMA (Preferred Reporting Items for Systematic Reviews and Meta-analyses) guidelines.

### 2.1. Search strategy

We systematically searched the Pubmed, Embase, and Cochrane libraries, Web of Science. Randomized controlled trials and cohort studies comparing the use of tirofiban and non-tirofiban (control) in patients undergoing EVT with anterior and posterior circulation acute stroke (published before March 22, 2022). Two review authors conducted independent literature searches and we used relevant combinations of terms: acute ischemic stroke (Mesh), acute brain infarction, acute cerebral infarction, stroke, stroke location, infarction location, anterior stroke, posterior stroke, anterior infraction, posterior infraction, infraction, cerebral vascular accident, brain vascular accident, combined with endovascular treatment (Mesh), endovascular therapy, endovascular procedures, endovascular techniques, thrombectomy, recanalization, angioplasty, stent, tirofiban (Mesh), tirofiban hydrochloride, tirofiban hydrochloride monohydrate. The citations generated from these searches were imported into the Zotero software and duplicate citations were removed. Subsequently, a systematic screening was carried out against the titles and keywords of the journals according to the inclusion and exclusion criteria developed below.

### 2.2. Selection criteria

#### 2.2.1. Inclusion criteria.

1. Clinical diagnosis of acute ischemic stroke.
2. Imaging (CTA or DSA) suggesting the presence of occlusion of a major artery.
3. The timely administration of endovascular treatment (EVT).
4. Data available from the literature for 2 treatment groups; The group with tirofiban; The group without tirofiban (control group).
5. Studies for anterior or posterior circulation acute stroke was analyzed.
6. The mode of administration of tirofiban: intravenous (IV) alone, arterial alone, arterial combined with IV.
7. Clinical randomized controlled trials or cohort studies.
8. Clear safety and efficacy outcome measures.

#### 2.2.2. Exclusion criteria.

1. Unfinished research projects.
2. Letters, reviews, newsletters, conference abstracts, descriptive studies, animal trials, case reports.
3. Studies with unextractable data, duplicate data, unclear descriptions.
4. Studies with <15 total cases.
5. Not differentiated for analysis of acute ischemic stroke in the anterior and posterior circulation.

### 2.3. Data extraction and quality assessment

Data were extracted independently by 2 recorders from studies that met the criteria and relevant disagreements were resolved through discussion.

The data extracted included: basic information, baseline characteristics, treatment regimen, location of occlusion, route, and dose of tirofiban administration, effectiveness outcomes, and safety outcomes. We contacted the corresponding authors by email to request further information where necessary. The Cochrane Risk of Bias Tool was used to evaluate the quality of RCTs, and the Newcastle-Ottawa Scale (NOS) was used to assess cohort studies with 8 items in 3 sections: study population selection, comparability between groups, and outcome measures, with NOS scores of ≥ 7 being classified as high-quality studies, 4 to 6 as moderate-quality studies, and 0 to 3 as low-quality studies. Overall, the RCTs in this meta were of high-quality and all cohort studies were of high-quality and achieved a good NOS score.

### 2.4. Outcome measures

Safety measures for the study: symptomatic intracranial hemorrhage, 3-month mortality. Symptomatic intracranial hemorrhage is defined by the European Cooperative Acute Stroke Study III. Study effectiveness measures: good functional outcome, excellent functional outcome, rate of recanalization. Good functional outcome was defined as an mRS score of 0 to 2 within 3 months postoperatively, excellent functional outcome was defined as an mRS score of 0 to 1 within 3 months postoperatively and recanalization was defined as a TICI (thrombolysis in cerebral infarction) score of 2b to 3.

### 2.5. Statistical analysis

Data were imported and analyzed by R (4.2.2) and Rev Manager, and statistical significance was defined as *P* < .05 if not otherwise stated. The Mantel–Haenszel method was used to calculate odd ratios (ORs) and 95% confidence intervals (CIs) for the random effects model with *I*^2^ > 50% and *P* ≤ .10 and the fixed effects model with *I*^2^ < 50% and *P* > .10. The sensitivity analysis was performed by sequentially removing studies 1 by 1 to assess the impact of each study on the overall results. Publication bias was detected by funnel plots, which we assessed for symmetry using Begg and Egger tests, and significant publication bias was defined as *P* < .1.

## 3. Results

### 3.1. Search results

**Figure 1.**
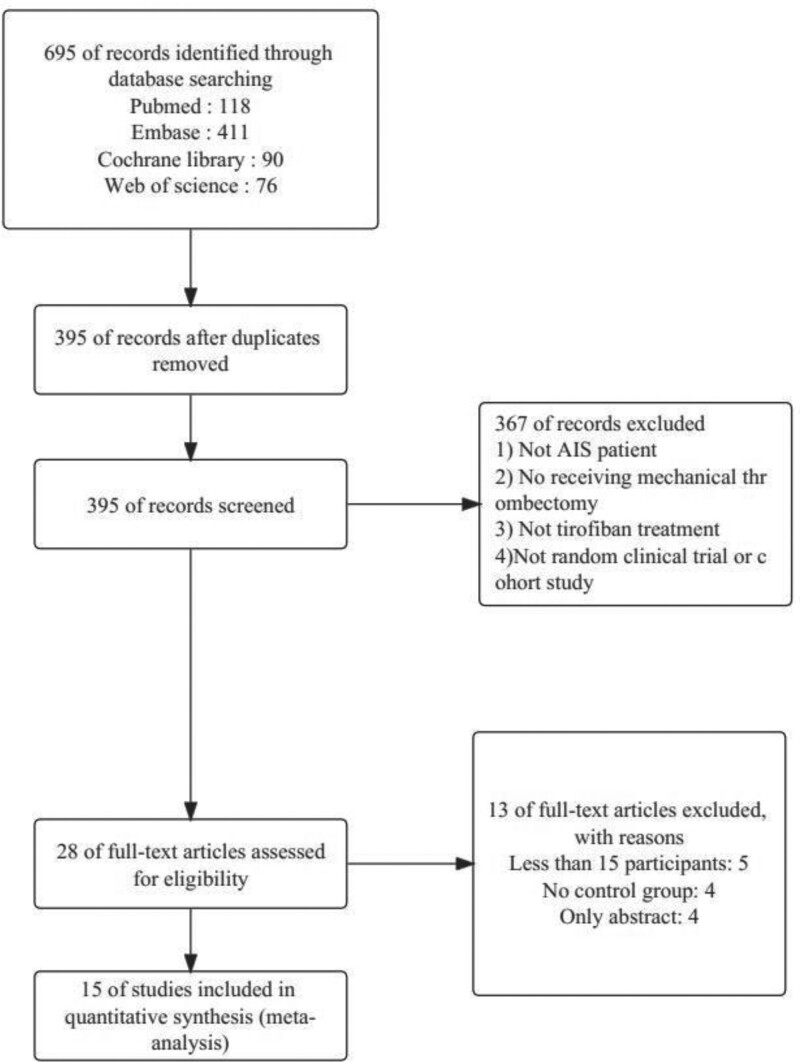
Flow chart of literature screening (specific screening paths and reasons for screening).

The initial literature search yielded a total of 695 studies. Based on the inclusion and exclusion criteria described above, 15 studies were carefully evaluated and screened for suitability for this meta-analysis. The specific literature selection process is illustrated in Figure 1. One study was a clinically randomized controlled study and the remaining 14 were observational cohort studies. Ten studies were from China, 2 studies were from Korea, 2 studies were from Germany and one was from Switzerland. Patients in all studies were treated with endovascular treatment and the use of tirofiban was recorded in detail. The base information for the studies is shown in Table 1 and the route of administration of tirofiban is shown in Table 2.

**Table 1:** Study characteristics and baseline information.

Author (yr)	Study center	Study design	Sample size (T/C)	Occlusion Location	EVT	Prior IVT, n (%)	NOS score
Baek et al^[17]^ (2021)	Single-center	Cohort	98 (30/68)	ACS (ICA/MCA)	MT + angioplasty/stenting	38.2	8
Jang et al^[18]^ (2021)	Multiple-center	Cohort	314 (35/279)	ACS (ICA/M1/M2)	MT/angioplasty/stenting	100	8
Gruber et al (2018)	Single-center	Cohort	32 (18/14)	ACS (ICA/M1)	MT + carotid stenting	100	7
Lee et al (2019)	Single-center	Cohort study	195 (60/135)	ACS (ICA/MCA)	MT + Stenting	84.1	8
Ma et al^[19]^ (2021)	Multiple-center	Cohort	201 (81/120)	ACS (ICA/M1)	MT/angioplasty/stenting	100	8
Qiu et al^[20]^ (2022)	Multiple-center	RCT	948 (463/485)	ACS (ICA/M1/M2)	MT/angioplasty/stenting	0	
Zhang et al^[21]^ (2019)	Multiple-center	Cohort	632 (154/478)	ACS (ICA/M1/M2)	MT	29.2	9
Zhong et al^[22]^ (2022)	Single-center	Cohort	145 (51/94)	ACS (ICA/M1/M2)	MT	19.9	8
Kellert et al^[23]^ (2013)	Single-center	Cohort	162 (50/112)	ACS + PCS	MT/stenting	73.3	8
Yang et al^[24]^ (2020)	Multiple-center	Cohort	662 (230/432)	ACS + PCS	MT/angioplasty/stenting	23.9	9
Zhao et al^[25]^ (2017)	Single-center	Cohort	180 (90/90)	ACS + PCS	MT/angioplasty/stenting	24	9
Chen et al^[26]^ (2022)	Multiple-center	Cohort	645 (363/282)	PCS (BA/VA)	MT/angioplasty/stenting	17.1	8
Pan et al^[27]^ (2022)	Multiple-center	Cohort	130 (64/66)	PCS (BA/VA)	MT/angioplasty/stenting	25	9
Quan et al^[28]^ (2019)	Multiple-center	Cohort	159 (85/74)	PCS (BA/VA)	MT/angioplasty/stenting	33	8
Sun et al^[29]^ (2020)	Multiple-center	Cohort	105 (74/31)	PCS (BA/VA)	MT/angioplasty/stenting	24.3	7

A high-quality study was defined as having a NOS ranking of 7–9.

ACS = anterior circulation stroke, BA = basilar artery, C = control group, EVT = endovascular treatment, ICA = internal carotid artery, IVT = intravenous thrombolysis, M1 = M1 segment of the middle cerebral artery, M2 = M2 segment of the middle cerebral artery, MT = mechanical thrombectomy, NOS = Newcastle-Ottawa Scale, NR = not reported, PCS = posterior circulation stroke, RCT = randomized controlled trial, T = tirofiban group, VA = vertebral artery.

**Table 2:** Route of administration of tirofiban.

Author (yr)	Comparation		IA	IV
Baek et al (2021)	No tirofiban	Post-MT	No	Continuous intravenous infusion (0.1 µg/kg/min) for 12 h
Jang et al (2021)	No tirofiban	Post-MT	An intra-arterial bolus (0.5 mg-1 mg)	No
Gruber et al (2018)	Aspirin (500 mg)	Pre-MT	An intra-arterial bolus (10 μg/kg)	Continuous intravenous fusion (9 μg/kg/h) for 60 h
Lee et al (2019)	No tirofiban	Post-MT	An intra-arterial bolus (0.125 mg)	Continuous intravenous infusions (6 μg/kg/h) for 12–24 h
Ma et al (2021)	No tirofiban	Post-MT	An intra-arterial bolus (0.25 mg-1 mg)	Continuous intravenous infusion (6 µg/kg/h) for 24 h
Qiu et al (2022)	Placebo	Pre-MT	No	An intravenous bolus (10 µg/kg) + Continuous intravenous infusion (9 µg/kg/h) for 24 h
Zhang et al (2019)	No tirofiban	Post-MT	An intra-arterial bolus (0.25 mg-1.0 mg)	No
Zhong et al (2022)	No tirofiban	Post-MT	No	An intravenous bolus (12 µg/kg) + Continuous intravenous infusion (6 µg/kg/h) for 24h
Kellert et al (2013)	No tirofiban	Post-MT	No	Local standard operating procedures recommend delivering tirofiban
Yang et al (2020)	No tirofiban	Post-MT	An intra-arterial bolus (0.25 mg-1 mg)	Continuous intravenous infusion (6 µg/kg/h) for 12 to 24 h
Zhao et al (2017)	No tirofiban	Post-MT	An intra-arterial bolus (0.25 mg-0.5 mg)	Continuous intravenous infusion (0.2–0.25 mg/h) for 12 to 24 h
Chen et al (2022)	No tirofiban	Post-MT	No	An intravenous bolus (12 μg/kg) + Continuous intravenous infusion (6 μg/kg/h) for 24 h
Pan et al (2022)	No tirofiban	Post-MT	An intra-arterial bolus (0.25–1 mg)	Continuous intravenously infusion (6–9 μg/kg/h) for 16–24 h
Quan et al (2019)	No tirofiban	Post-MT	No	Continuous intravenous tirofiban infusion (0.2–0.25 mg/h) for 12–24 h
Sun et al (2020)	No tirofiban	Post-MT	No	Continuous Intravenous tirofiban infusion (9 µg/kg/h) for 24 h

IA = intra-arterial, IV = intravenous, MT = mechanical thrombectomy.

### 3.2. Safety outcomes

#### 3.2.1. sICH.

**Figure 2.**
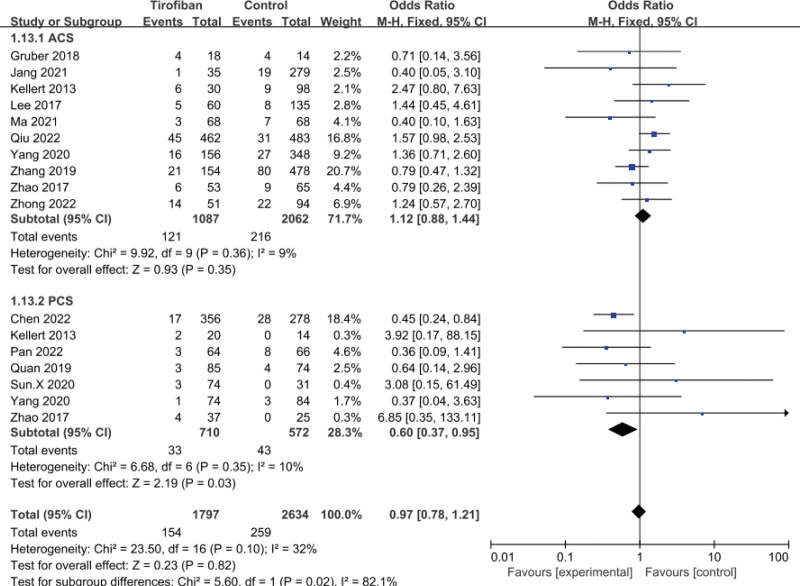
Forest plot and meta-analysis of the risk of sICH. sICH = symptomatic intracranial hemorrhage.

A total of 14 studies were included in the pooled analysis of sICH. It was found that tirofiban did not significantly increase the rate of sICH (OR = 0.97, 95% CI = 0.78–1.21, *P* = .35; Fig. 2). For the anterior circulation stroke (ACS) subgroup, there was no statistically significant difference in the incidence of sICH between the tirofiban and control groups (OR = 1.12, 95% CI = 0.88–1.44, *P* = .35; Fig. 2). In contrast, in the posterior circulation stroke (PCS) subgroup, tirofiban significantly reduced the incidence of sICH in patients (OR = 0.60, 95% CI = 0.37–0.95, *P* = .03; Fig. 2). Significant heterogeneity was seen for both subgroups (*P* = .36, *I*^2^ = 9%; *P* = .35, *I*^2^ = 10%; Fig. 2).

### 3.3. Three-month mortality

**Figure 3.**
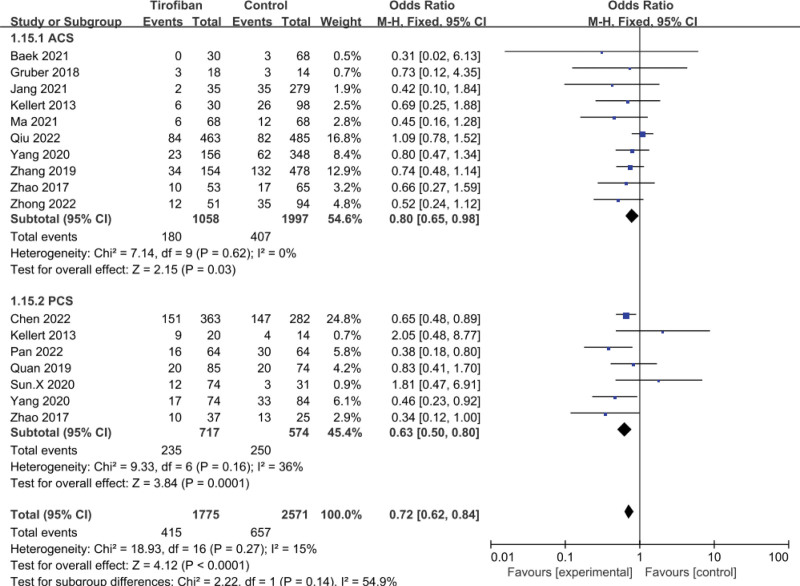
Forest plot and meta-analysis of mortality at 3 months.

A total of 14 studies were included in the safety analysis on mortality at 3 months after EVT and showed that tirofiban significantly reduced mortality at 3 months (OR = 0.72, 95% CI = 0.62–0.84, *P* < .0001; Fig. 3). The results showed that in the ACS subgroup, tirofiban significantly reduced postoperative mortality at 3 months (OR = 0.80, 95% CI = 0.65-0.98, *P* = .03; Fig. 3) and no significant heterogeneity was seen (*P* = .62, *I*^2^ = 0%; Fig 3). In the PCS subgroup, the tirofiban group also had a lower mortality rate at 3 months postoperatively than the control group (OR = 0.63, 95% CI = 0.50-0.80, *P* = .0001; Fig. 3), with mild heterogeneity (*P* = .16, *I*^2^ = 36%; Fig 3).

### 3.4. Effective outcomes

#### 3.4.1. Good functional outcome.

**Figure 4.**
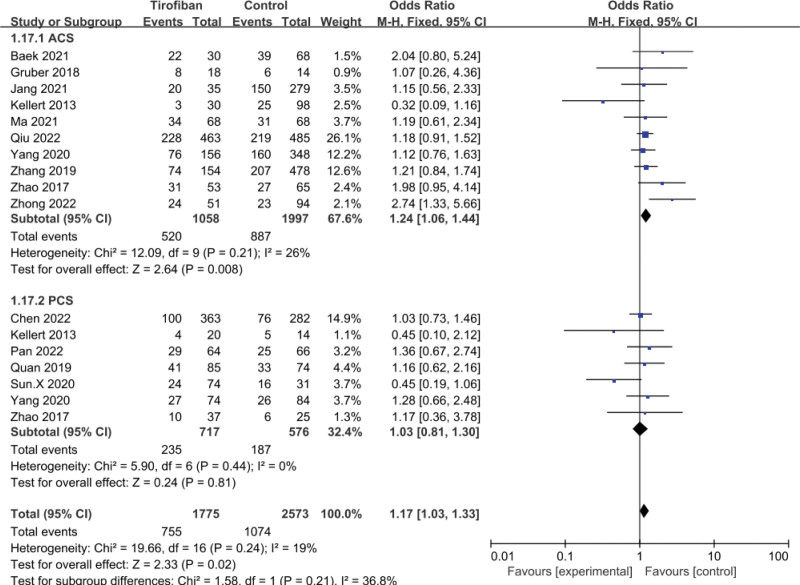
Forest plot and meta-analysis of 3-month mRS 0–2 score.

A total of 14 studies were included in the pooled analysis and showed that tirofiban was effective in improving the functional outcome of patients with AIS thrombectomy (OR = 1.17, 95% CI = 1.03–1.33, *P* = .02; Fig. 4). In patients with ACS, the tirofiban group had better functional outcome than the control group (OR = 1.24, 95% CI = 1.06–1.44, *P* = .008; Fig. 4), with mild heterogeneity (*P* = .21, *I*^2^ = 26%; Fig. 4). Notably, in the subgroup of PCS, there was no statistically significant difference between the tirofiban and control group (OR = 1.03, 95% CI = 0.81–1.30, *P* = .81; Fig. 4), and no significant heterogeneity was seen (*P* = .44, *I*^2^ = 0%; Fig. 4). Sensitivity analysis showed that *I*^2^ was reduced to 0 after removing the Kellert et al^[21]^ or Zhong et al^[22]^ studies, with both studies being the main source of heterogeneity.

### 3.5. Excellent functional outcome

**Figure 5.**
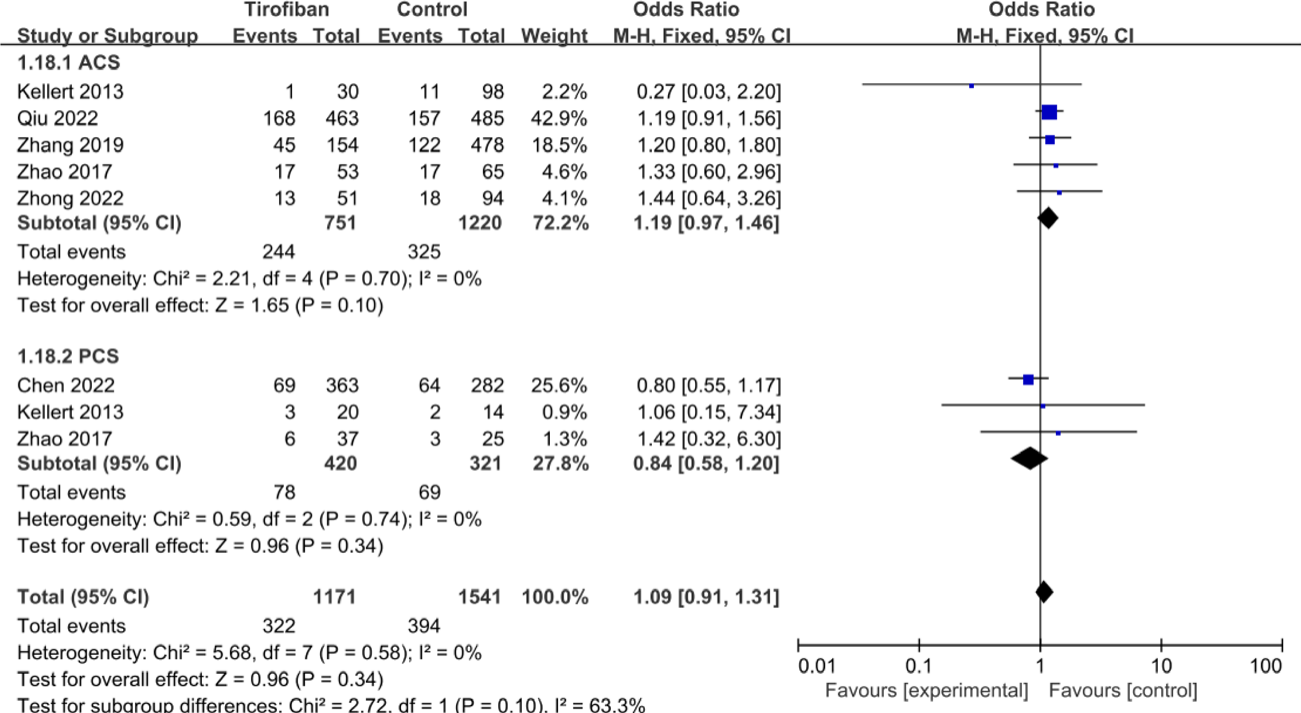
Forest plot and meta-analysis of 3-month mRS 0–1 score.

A total of 6 studies were included in the pooled analysis. No statistically significant differences were found for the tirofiban group compared to the control group in either the ACS or PCS subgroups (*P* = .10; *P* = .34, Fig. 5). However, in terms of OR, the 2 subgroups showed opposite trends. In the ACS group of patients, excellent functional outcome was superior in the tirofiban group than in the control group (OR = 1.19, 95% CI = 0.97–1.46, *P* = .10; Fig. 5). In PCS patients, the opposite conclusion was reached, with the control group having a superior functional regression to the tirofiban group (OR = 0.84, 95% CI = 0.58–1.20, *P* = .34; Fig. 5). No significant heterogeneity was seen in the study (*P* = .58, *I*^2^ = 0; Fig. 5).

### 3.6. Recanalization rate

**Figure 6.**
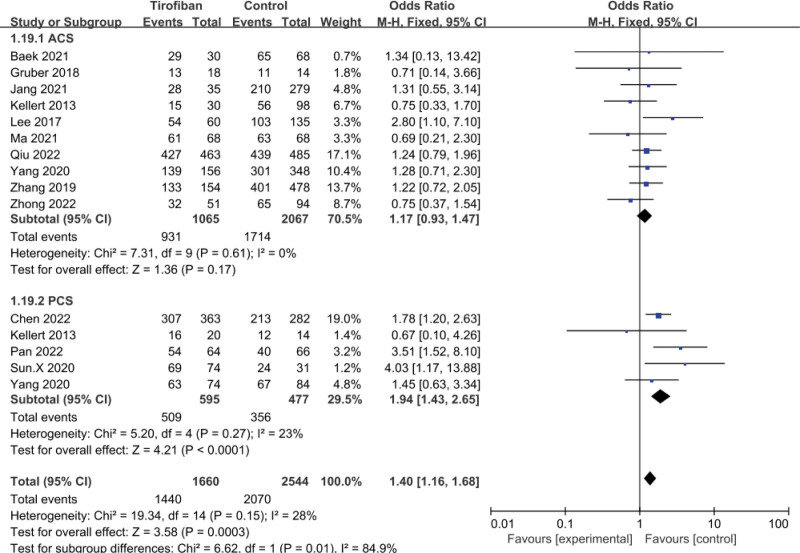
Forest plot and meta-analysis of recanalization.

A total of 13 studies were included in the pooled analysis of recanalization rates, suggesting that tirofiban was effective in improving recanalization (OR = 1.40, 95% CI = 1.16–1.68, *P* = .0003; Fig. 6). The results of the ACS subgroup analysis suggested that there was no significant difference in recanalization rates between the tirofiban and control groups (OR = 1.17, 95% CI = 0.93–1.47, *P* = .17; Fig. 6). In contrast, in the PCS group, there was a significant difference in arterial recanalization between the tirofiban group and the control group (OR = 1.94, 95% CI = 1.43–2.65, *P* < .0001; Fig. 6). There was mild heterogeneity in the PCS subgroup (*P* = .27, *I*^2^ = 23%; Fig. 6). Sensitivity analysis showed that after the removal of the Pan et al^[27]^ study, the *I*^2^ value decreased to 0, suggesting a major source of heterogeneity.

### 3.7. Sensitivity analysis

We conducted the sensitivity analysis in 2 ways: Changing the analytical model to a random effects model, with results consistent with the preliminary analysis (Figure S1, Supplemental Digital Content, http://links.lww.com/MD/J799); Individual studies were removed sequentially using the R software (4.4.2). Based on the sensitivity analysis (Figure S2, Supplemental Digital Content, http://links.lww.com/MD/J801), no statistically significant difference was seen between the tirofiban and control groups after the removal of Chen et al^[26]^ in the pooled analysis of sICH rates (OR = 0.87, 95% CI = 0.43–1.78, *P* = .70). Other sensitivity analysis results were consistent with the preliminary analysis.

### 3.8. Publication bias

Regarding the results of the funnel plot analysis, it can be seen that there is no significant asymmetry in the shape of the funnel plot from the visible part (Figure S3, Supplemental Digital Content, http://links.lww.com/MD/J802). However, the points corresponding to each study in the funnel plot are unevenly distributed, mainly in the 3 indicators of sICH, good functional outcome, and recanalization rate.

## 4. Discussion

We conducted a meta-analysis of the efficacy and safety of tirofiban in stroke patients undergoing EVT by including 15 studies with a total of 4608 patients, with a subgroup analysis of embolization sites. In patients with anterior circulation stroke, tirofiban significantly reduced 3-month mortality without increasing symptomatic intracranial hemorrhage and significantly improved good functional outcome, but did not significantly improve recanalization rate or excellent functional outcome. In patients with posterior circulation stroke, tirofiban significantly reduced 3-month mortality and symptomatic intracranial hemorrhage, and significantly improved recanalization rate, but did not significantly improve good and excellent functional outcome.

The safety and efficacy of endovascular treatment for anterior circulation stroke have been demonstrated in several large multicentre clinical studies over several years, but there is some debate as to whether EVT should be performed in patients with vertebrobasilar stroke. Fortunately, recent studies such as the Baoche study^[12]^ and the attention study^[13]^ have confirmed the feasibility of EVT in basilar artery stroke, providing high-level evidence-based evidence for the development of clinical guidelines for the endovascular treatment of large artery occlusions in the vertebrobasilar artery. Differences in thrombus type, clinical presentation, and perioperative management between the anterior and posterior circulation affect the efficacy of tirofiban in patients with acute ischemic stroke after the operation.

**Figure 7.**
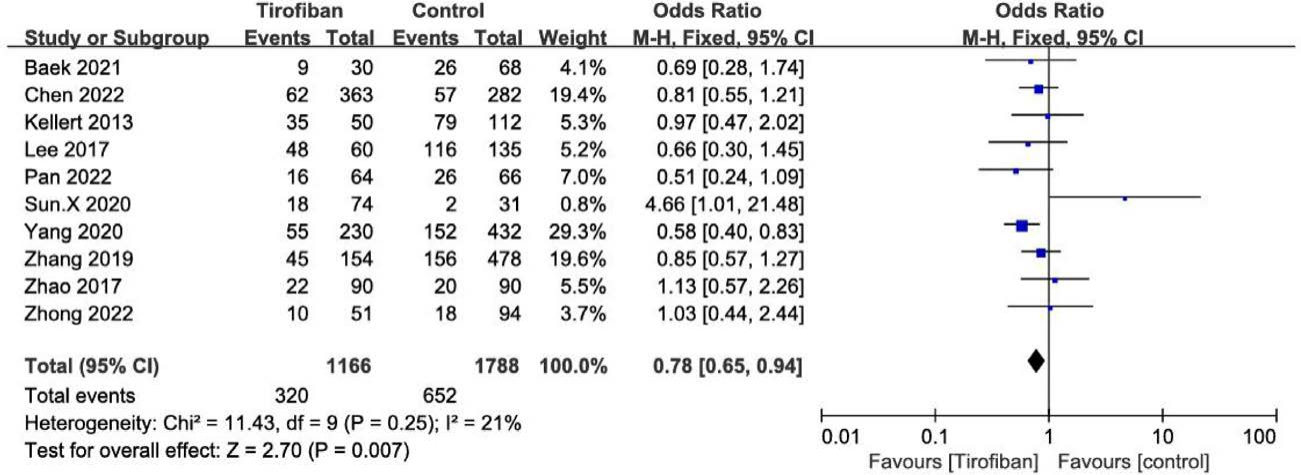
Forest plot and meta-analysis of intravenous thrombolysis.

The safety of tirofiban, a highly selective GP IIb/IIIa receptor antagonist with a rapid onset of action and short half-life, has been effectively demonstrated in coronary interventions, but its utilization in patients with AIS undergoing EVT is controversial because of the risk of hemorrhagic transformation in patients with acute ischemic stroke undergoing EVT. In our study, the tirofiban group had a lower sICH rate compared with the control group, although this was not statistically different. Previous correlation meta-analyses^[30,31]^ have also reached the same conclusion. The reasons for this are, firstly, that large-vessel occlusive plaque in the atherosclerotic form is unstable and prone to rupture after manipulation leading to distal vessel embolism and hemorrhagic transformation, which is effectively reduced by fast-acting tirofiban. Secondly, the higher rate of IV thrombolysis in the control group increased the incidence of symptomatic intracranial hemorrhage to some extent (OR = 0.78, 95% CI = 0.65–0.94, *P* = .007; Fig. 7). Third, previous studies have suggested that tirofiban has a better safety profile and lower risk of hemorrhage in patients with large atherosclerotic stroke compared with cardiogenic stroke. In 2 clinical studies of the posterior circulation, Chen et al^[26]^ and Pan et al,^[27]^ the tirofiban group had a significantly higher proportion of large artery atherosclerotic strokes. This was also the reason for the significantly lower rate of sICH in the tirofiban group in the posterior circulation subgroup.

A series of Meta-analyses have shown^[16,32,33]^ that endovascular therapy combined with adjuvant tirofiban in stroke patients is effective in reducing mortality and functional outcome at 3 months postoperatively and does not increase the rate of symptomatic intracranial hemorrhage in patients. However, the results of past meta-studies have been inconsistent concerning effectiveness indicators. Interestingly, in our study, tirofiban had a significantly higher clinical prognosis for anterior circulation stroke, but no significant improvement in recanalization rates. We believe the reasons for this are twofold: First, Kellert et al^[23]^ conducted their research almost a decade ago. The majority of other trials employed second-generation retrieval stents, but not Kellert et al,^[23]^ who used different endovascular retrieval procedures in around 20% of patients (e.g., balloon expansion technique, merci retriever, and stent implantation). As a result, there were lower recanalizations and higher sICH rates. Second, the 6 to 24 hour treatment time window in the Qiu et al^[20]^ was assessed by the aspects score on CT scans, but many of the hospitals involved did not have the relevant analysis software and there may have been more significant variations in CT perfusion assessment of ischemic core and semidark zone volumes, leading to bias in the included patients and higher rate of symptomatic intracranial hemorrhage.

Previous studies have suggested that tirofiban has the potential to improve functional prognosis by reducing inflammatory factors,^[34–37]^ protecting the vascular endothelium,^[38]^ and slowing the progression of atherosclerosis.^[39,40]^ In general, tirofiban significantly increased the rate of arterial recanalization after thrombectomy in patients with AIS and improved the good functional prognosis of patients. However, in subgroup analyses, it did not significantly increase the rate of arterial recanalization in patients with ACS and improve the good functional outcome of patients with PCS. There are several reasons for the significant difference between the subgroups: On arterial recanalization, firstly, there was a higher proportion of large atherosclerotic strokes in the PCS group, and tirofiban had a better thrombogenic effect against white thrombi, resulting in a higher rate of arterial recanalization. As a comparison, the proportion of cardiac strokes was significantly higher in the ACS group, and fibrin-rich red thrombi reduced the effectiveness of tirofiban in recanalizing arteries. Secondly, studies have reported that patients with vertebrobasilar infarction have better rates of collateral circulation and better rates of revascularization because large artery atherosclerotic stroke is a relatively long-forming process.^[41]^ Thirdly, compared with the PCS group in which patients were given tirofiban after thrombectomy, the ACS group was set up with tirofiban before thrombectomy by Gruber et al and Qiu et al This did not have an effective antithrombotic effect on re-embolism after thrombectomy, and therefore did not have a higher rate of recanalization. In terms of functional prognosis, on the 1 hand, patients with anterior circulation stroke have significant motor and speech-related symptoms and are more likely to be evaluated in time for emergency thrombolysis and thrombectomy. Posterior circulation strokes can accumulate in the brainstem, and symptoms are often atypical, leading to misdiagnosis and underdiagnosis, and a higher proportion of critical illnesses, so that the traditional time window for retrieval is exceeded by the time of diagnosis. The time window for thrombolysis is exceeded by the time of diagnosis. On the other hand, it is clear from our included studies that patients with posterior circulation strokes are more likely to receive other remedial treatments (e.g., stenting or angioplasty treatment), which increases the rate of recanalization of the patient’s arteries but prolongs the operative time of the whole procedure and additionally increases the risk of endothelial damage.

The GP Iib/IIIa receptor antagonists commonly used today are abciximab, eptifibatide, and tirofiban. Abciximab binds irreversibly to the GPIIb/IIIa receptor and has a relatively long duration of action. Two thousand eighteen guidelines published by the AHA/ASA state that abciximab may carry a higher risk of bleeding in patients with AIS and is not recommended and is currently used less frequently in clinical practice.^[42]^ Compared to tirofiban, there are almost no reports of eptifibatide being used for thrombectomy. For patients undergoing thrombectomy, Ma et al^[43]^ found that eptifibatide significantly improved recanalization rates and clinical functional prognosis without increasing the rate of sICH. In addition, analysis in the study found no difference in prognosis between anterior and posterior circulation strokes. As a highly selective antagonist of GP IIb/IIIa receptors, eptifibatide has greater targeting and specificity, is more selective, has a faster onset of action, and has a stronger inhibitory effect on platelet aggregation. In our study, we found that tirofiban did not significantly improve the functional prognosis of patients with vertebrobasilar artery thrombosis, whereas the results from Ma study suggest that it may be a suitable adjunct to posterior circulation thrombosis for improving prognosis and revascularization in posterior circulation stroke thrombosis.

## 5. Limitations

Our study had the following limitations: firstly, the included studies did not have specific rules for the mode of administration and dose of tirofiban. The effects of arterial pump administration and continuous IV infusion administration on outcome events need to be further investigated. Second, the rate of IV thrombolysis prior to endovascular takedown varied considerably, as in the Jang et al^[18]^ and Ma et al^[19]^ studies where patients were treated with bridging therapy, the prognostic impact of which cannot be ignored. Thirdly, there was mild heterogeneity in the study, as shown by the uneven distribution of the corresponding points in the funnel plot. Our study analyzed the sources of heterogeneity by analyzing the subgroups and analyzing the reasons for the heterogeneity. Fourthly, there is a lack of multicentre randomized controlled trials on the efficacy of tirofiban in patients with posterior circulation large artery occlusive stroke undergoing EVT, so there is a lack of high-level evidence-based evidence in the PCS subgroup, and future RCT studies are expected.

## 6. Conclusion

In patients with acute stroke in the anterior circulation treated endovascularly, tirofiban significantly reduced 3-month mortality and improved good functional outcome, but did not increase sICH rates or significantly improve the excellent outcome. In patients with acute stroke in the posterior circulation treated with endovascular therapy, tirofiban improved arterial recanalization rates, reduced sICH rates, and 3-month mortality, but did not significantly improve good functional outcome. Tirofiban is safe and effective in both anterior and posterior circulation stroke patients undergoing EVT. More multicentre randomized controlled studies are needed for the future of stroke-related antiplatelet aggregation therapy for different embolic sites.

## Author contributions

**Conceptualization:** Chen Liu, Mingsu Liu, Guangqin Li.

**Data curation:** Chen Liu, Xun Yang, Jinping Wang.

**Formal analysis:** Chen Liu.

**Funding acquisition:** Jinping Wang, Guangqin Li.

**Investigation:** Xun Yang, Mingsu Liu.

**Methodology:** Chen Liu, Xun Yang.

**Software:** Chen Liu, Mingsu Liu.

**Supervision:** Guangqin Li.

**Validation:** Guangqin Li.

**Visualization:** Guangqin Li.

**Writing – original draft:** Chen Liu, Xun Yang.

**Writing – review & editing:** Chen Liu, Mingsu Liu, Guangqin Li.

## Supplementary Material
