# Supplementary material for: Systematic review and meta-analysis of the efficacy and safety of adjunctive use of tirofiban in patients treated with endovascular therapy for acute ischemic stroke at different embolic sites
Source: Medicine (Baltimore). 2023 Oct 6;102(40):e35091. doi: 10.1097/MD.0000000000035091 (PMC10553052; doi:10.1097/MD.0000000000035091)
Supplement: Supplementary file 1 [file medi-102-e35091-s001.docx]

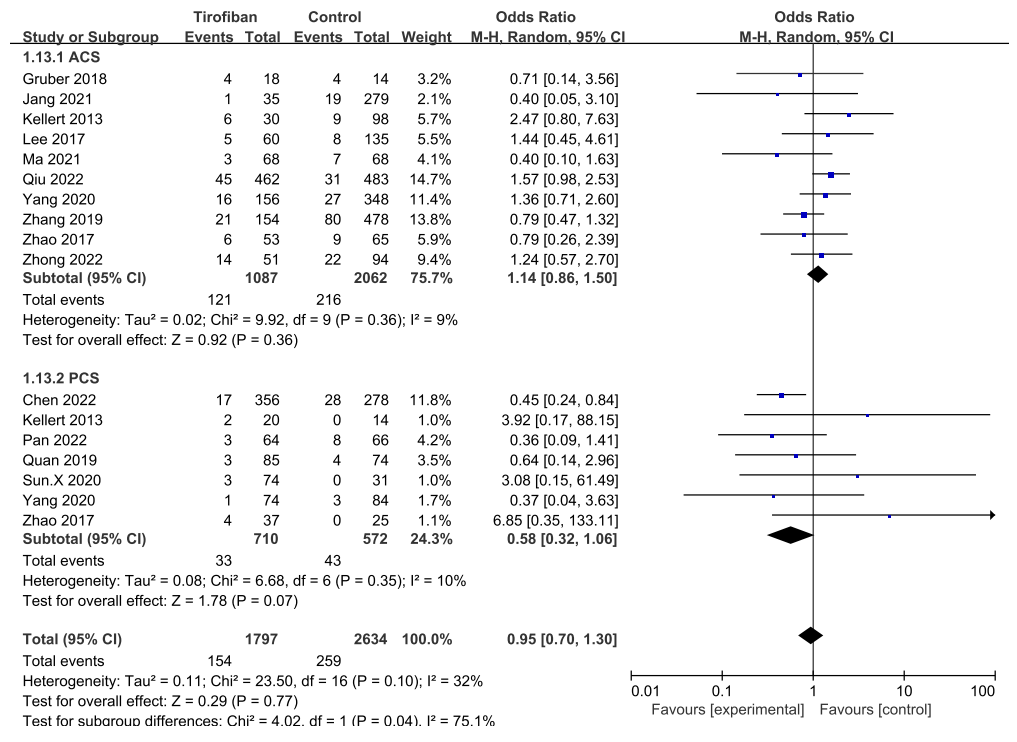


**Forest plot and meta-analysis of the risk of sICH(Random Effect)**


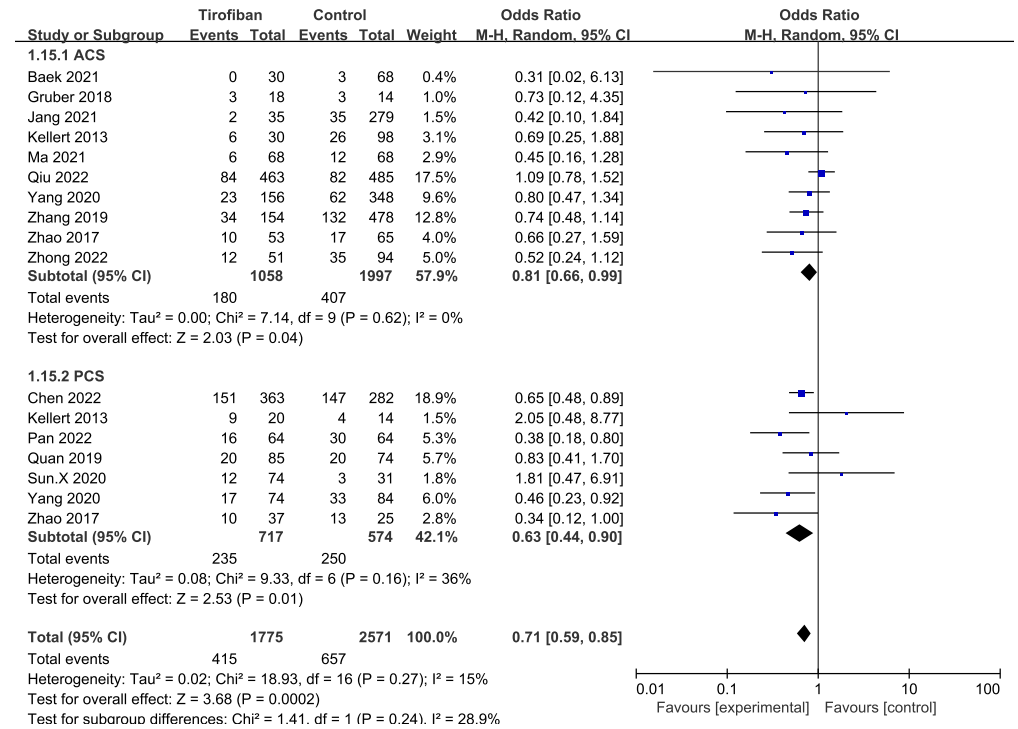


**Forest plot and meta-analysis of mortality at 3 months(RE)**


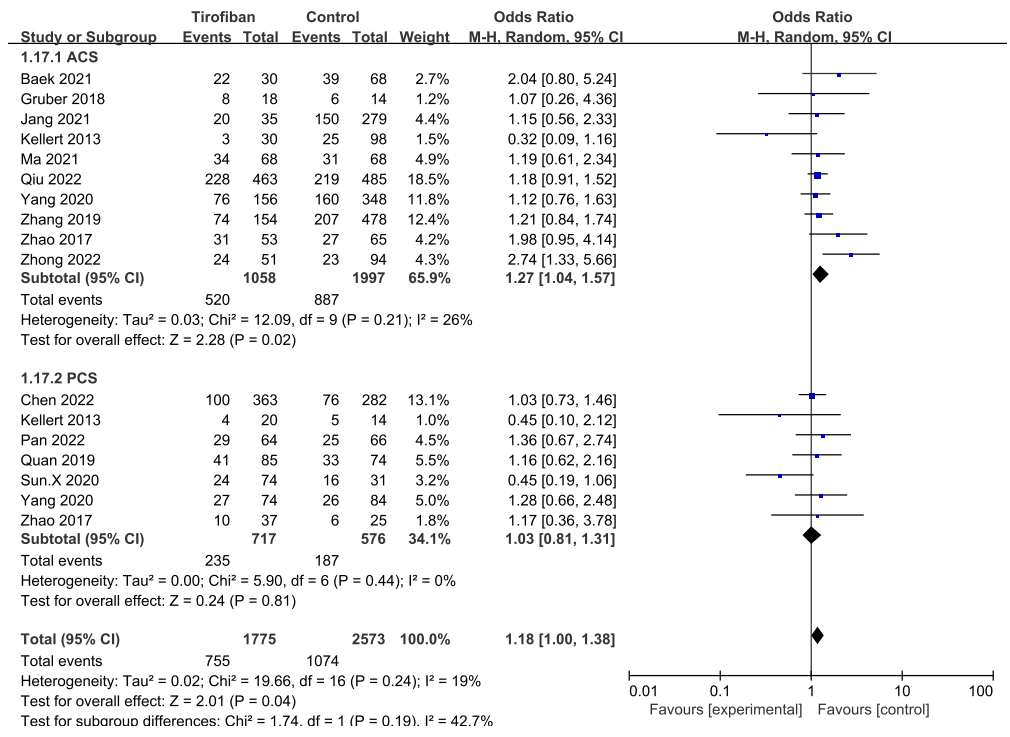


**Forest plot and meta-analysis of mRS 0–2(RE)**


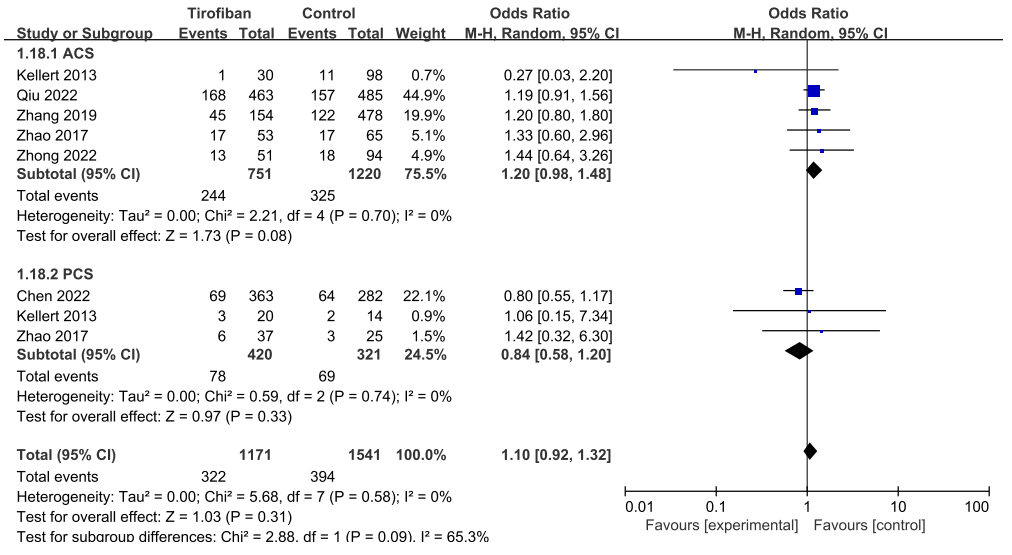


**Forest plot and meta-analysis of mRS 0–1(RE)**


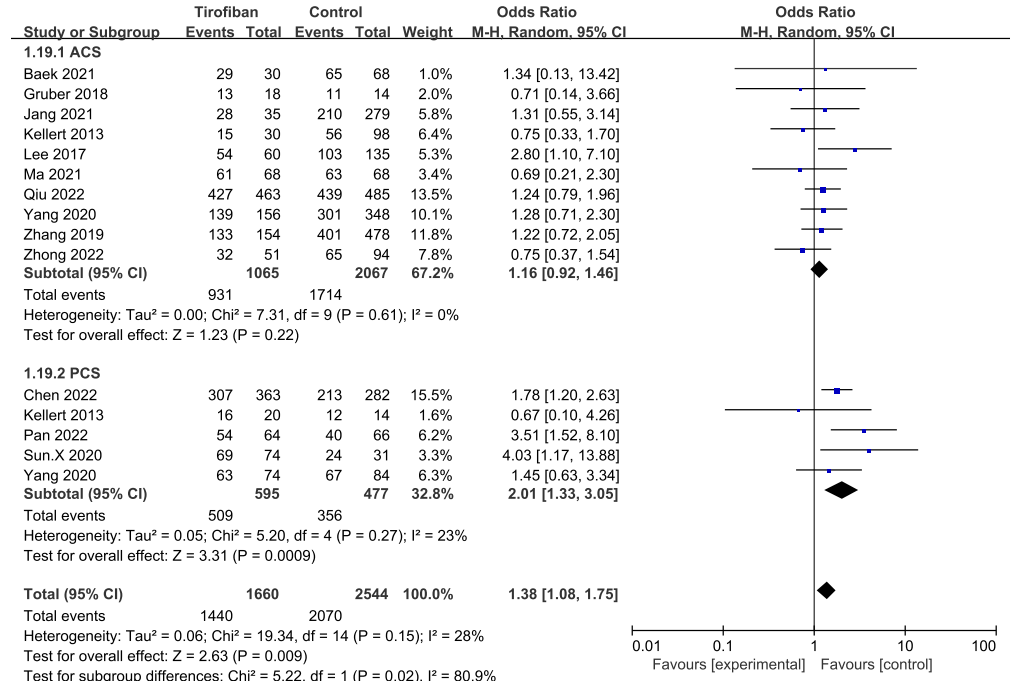


**Forest plot and meta-analysis of recanalization rate(RE)**
