# Supplementary material for: Systematic review and meta-analysis of the efficacy and safety of adjunctive use of tirofiban in patients treated with endovascular therapy for acute ischemic stroke at different embolic sites
Source: Medicine (Baltimore). 2023 Oct 6;102(40):e35091. doi: 10.1097/MD.0000000000035091 (PMC10553052; doi:10.1097/MD.0000000000035091)
Supplement: Supplementary file 2 [file medi-102-e35091-s002.docx]

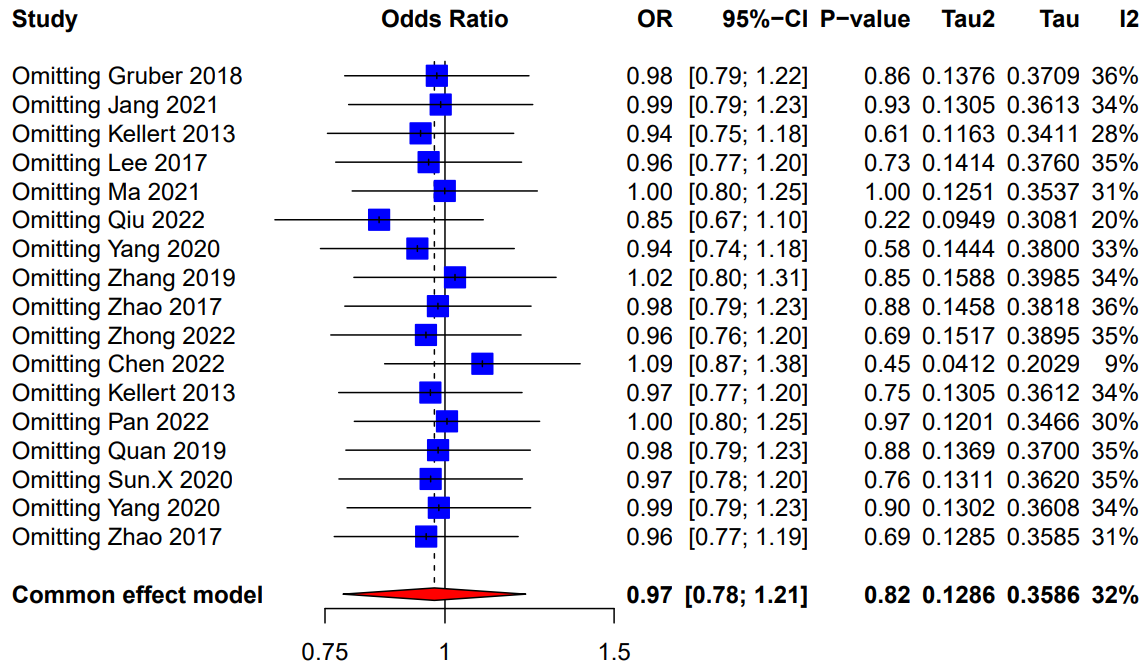


**Sensitive analysis of sICH**


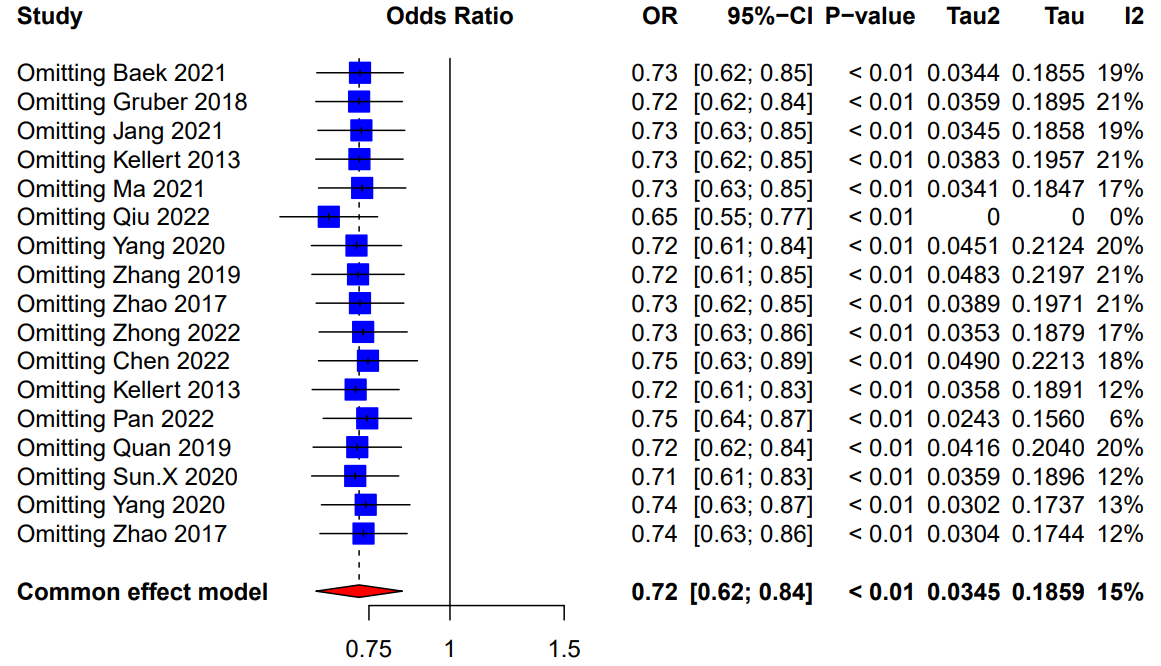


**Sensitive analysis of 3-month mortality**


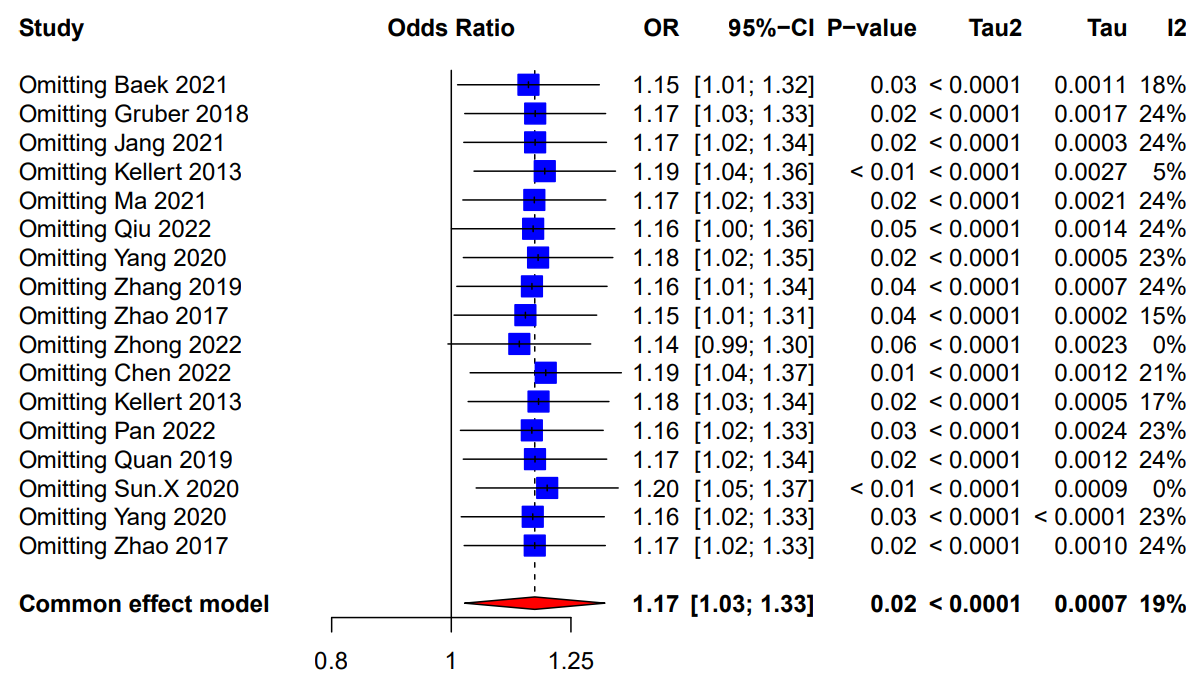


**Sensitive analysis of mRS 0-2**


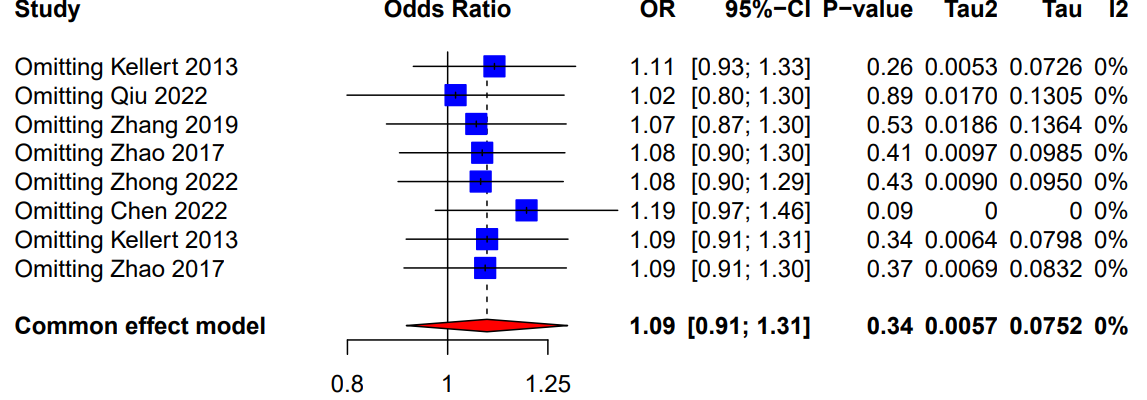


**Sensitive analysis of mRS 0-1**


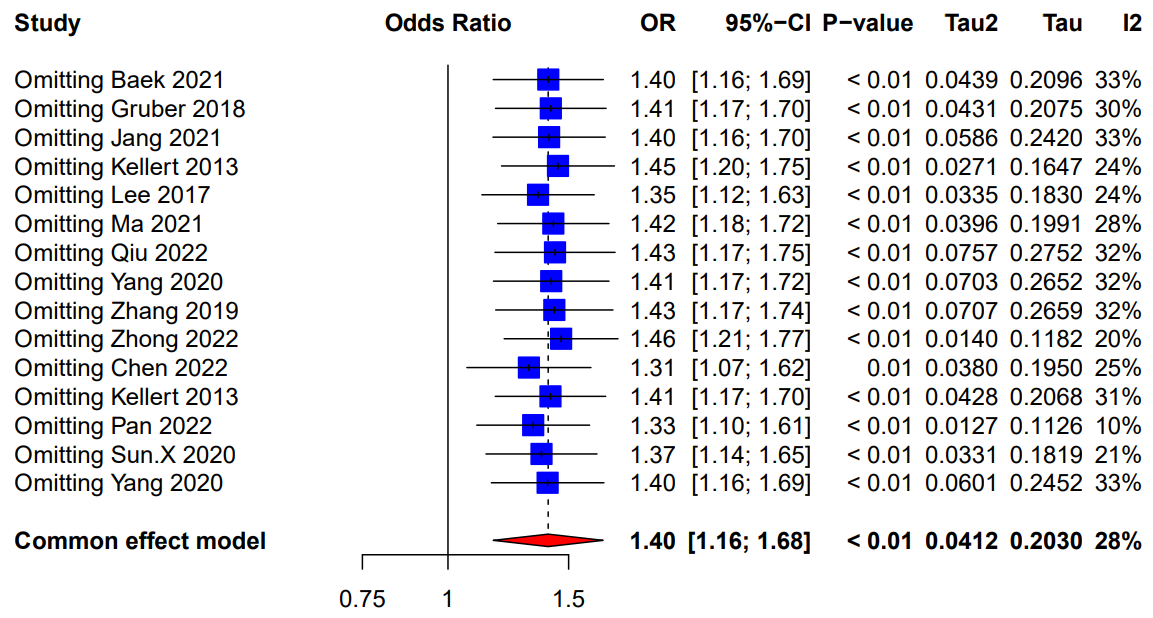


**Sensitive analysis of recanalization**


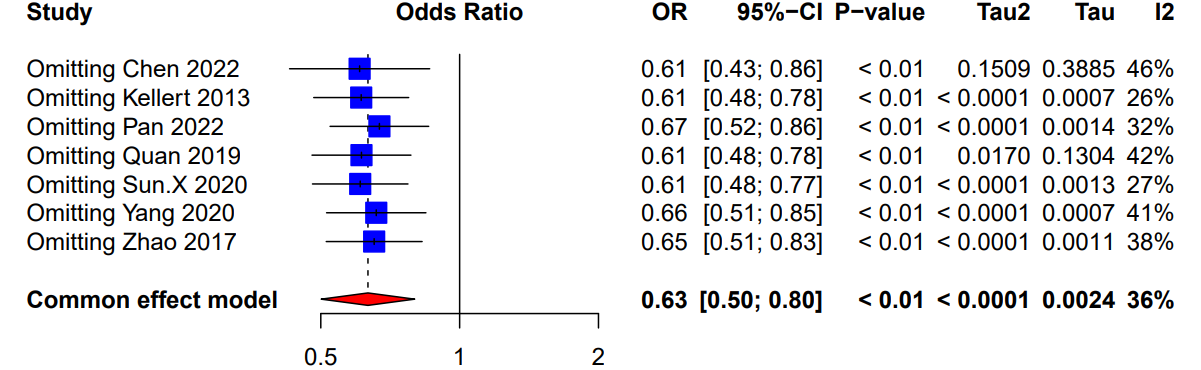


**Sensitive analysis of 3-month mortality of PCS**


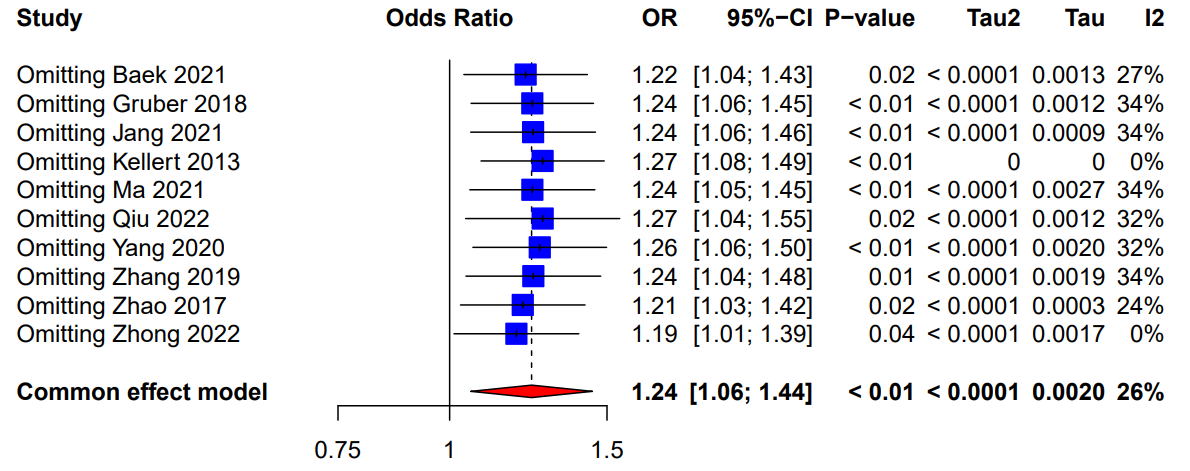


**Sensitive analysis of mRS 0-2 of ACS**


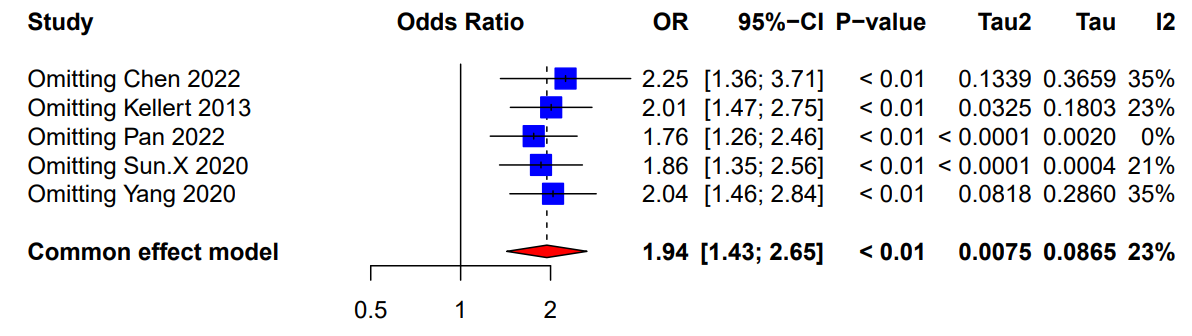


**Sensitive analysis of recanalization of PCS**
